# The innovative development in interferon beta treatments of relapsing‐remitting multiple sclerosis

**DOI:** 10.1002/brb3.696

**Published:** 2017-05-08

**Authors:** Claus Madsen

**Affiliations:** ^1^ Department of NeurologyOdense University HospitalOdense CDenmark

**Keywords:** interferon beta, multiple sclerosis, peginterferon beta‐1a, RRMS

## Abstract

The introduction of interferon beta therapies more than 20 years ago marked a milestone in the treatment of relapsing‐remitting multiple sclerosis (RRMS) with a significant impact on the approach to modern multiple sclerosis (MS) care. Key learnings and perspectives from the early days of disease modifying therapies in MS have improved the knowledge base of MS, need for treatment, and patient care. The continuous development of interferons over the past two decades outlines a journey with increased understanding of the pharmacodynamics and pharmacokinetic mechanisms of interferons, leading to innovative formulations with an improved benefit/risk profile.

## 1 Introduction

Multiple sclerosis (MS) is the most common chronic cause of neurological disability among younger adults (Compston & Coles, 2008; Noseworthy, Lucchinetti, Rodriguez, & Weinshenker, 2000). Acknowledging that until 20 some years ago the treatment of MS patients focused mainly on treating relapses with systemic corticosteroids and providing various symptomatic treatment, the introduction of interferon beta therapies in 1993 (interferon beta‐1b) and in 1996 (interferon beta‐1a) fundamentally changed the MS treatment paradigm. For patients, treatment transferred from symptomatic management into disease modifying long‐term therapy, and for neurologists their roles changed from passively observing and awaiting patient worsening into proactively treating the underlying disease in the MS patients. This implied logistical changes to MS care in general with the need for improved follow‐up.

The interferon betas represent the first class of disease modifying therapies (DMTs) for MS and have contributed considerably to the understanding of the immunomodulatory mechanisms in MS. Since then several other DMTs have been approved for the treatment of MS. However, with a growing body of evidence on the long‐term benefits by reduction of disability progression (Scalfari et al., 2010; Trojano et al., 2009), reduced mortality, and data to suggest maternal and fetal relative safety in pregnancy outcomes (Amato & Portaccio, 2015; Romero, Lunzmann, & Bugge, 2015) — interferons maintain an important role in the treatment of RRMS. Furthermore, the interferon betas are acknowledged as standard of care and have recently acted as an active comparator in two different large scale prospective randomized controlled clinical trials testing new MS therapies (Hauser et al., 2017; Kappos, Wiendl, et al., 2015).

The recent introduction of an innovative formulation of interferon beta‐1a has improved patient convenience by a markedly reduction in number of injections, a favorable risk profile building on two decades of clinical use, and potentially improved efficacy (Calabresi et al., 2014; Kieseier et al., 2014). The purpose of this paper is to give a historic overview of interferon treatment in MS, marking the continued development of the drug class for more than two decades after the initial introduction and acknowledging the history as well as part of the future in MS treatment.

## 2 Paradigm Shift in the Treatment of MS

In patients with RRMS with preserved walking function and with signs of disease activity, demonstrated by relapses over the prior 1 to 2‐year period, interferon beta therapies have been shown to reduce the number of relapses by an average of 30% compared to placebo and in particular the number of severe relapses is reduced (Calabresi et al., 2014; Jacobs et al., 1996; Kieseier et al., 2014; PRISMS Study Group, 1998; Rudick et al., 1997; The IFNB Multiple Sclerosis Study Group, 1993). Furthermore, interferon beta delays the development of permanent neurological disability and disease activity evaluated by magnetic resonance imaging (MRI). Patients with the first suspected demyelinating episode and typical MS‐changes on MRI or less typical changes on MRI combined with oligoclonal bands are at high risk of developing clinically definite MS (CDMS) over the following period of 2 years (Polman et al., 2005). Treatment with interferon beta prolongs the time to CDMS and reduces relapse rate and disease progression (Kappos, Kuhle, et al., 2015). The introduction of interferon betas for the treatment of RRMS represented a paradigm shift in the management of MS and fueled a significant interest from the patient community. At that time the criteria for interferon beta therapy required patients to have accumulated a certain amount of disability to be eligible for treatment. However, data have since then proven the benefit of early initiated interferon treatment (Kappos, Kuhle, et al., 2015), exposing the rigid treatment criteria in the late 1990s to be scientifically unjustified, however, somehow rationalized through clinical experience. Retrospectively, it proved itself to be a clinical paradox that the window of opportunity for obtaining maximum benefit from the DMT was narrowed considerably over time. On one hand a patient with little or no current disease activity (i. e., relapse) was ineligible to receive interferon beta, however, after subsequently experiencing disability progression following a relapse, the patient was at risk of having missed the opportunity for receiving disease modifying treatment. Fortunately, treatment with interferon beta is initiated earlier (Kappos, Kuhle, et al., 2015).

### 2.1 Reorganizing MS outpatient care

The introduction of interferon beta therapies called for a reorganization of the outpatient care in MS. In Denmark, biannual follow‐up visits were introduced in the out‐patient MS clinics. Before the reorganization, outpatient care and follow‐up were determined by the occurrence of relapses and need for symptomatic treatment only. With the introduction of DMTs, continuous and more frequent follow‐up was also generally introduced, improving the overall care, nursing, and clinical follow‐up. Today the follow‐up introduced in the mid‐1990s has become the standard at many MS clinics. Treatment with interferon beta should be followed by clinical examination and blood tests including hemoglobin, leukocytes with differential count, liver enzymes, serum creatinine, and electrolytes after 3 and 6 months, and then every 6 months. Measurement of neutralizing antibodies should be performed every 6 months for the first 2 years of treatment. In the event that all tests for neutralizing antibodies during this period have been negative, monitoring may stop, as the probability of developing neutralizing antibodies after this time is minute (Sorensen, Koch‐Henriksen, Ross, Clemmesen, & Bendtzen, 2005). Signs of disease activity should reactivate monitoring of neutralizing antibodies. Patients should be clinically evaluated after 2 years of treatment, and long‐term treatment should be decided on an individual basis by the treating physician. Treatment should be discontinued if the patient develops chronic progressive MS (Sorensen et al., 2005).

### 2.2 Pharmacodynamic properties of interferons in MS

Interferons are a family of naturally occurring proteins that are produced by eukaryotic cells in response to viral infection and other biological inducers. Interferons are cytokines that mediate antiviral, anti‐proliferative, and immunomodulatory activities. Three major forms of interferons have been distinguished: alpha, beta, and gamma. Interferons alpha and beta are classified as Type I interferons and interferon gamma is a Type II interferon. These three types of interferons have overlapping but clearly distinguishable biological activities. Notably the systemic administration of gamma interferon has pronounced effects on cellular immunity in MS and on disease activity within the CNS, suggesting that the attacks induced during treatment with interferon gamma are immunologically mediated, leaving interferon gamma unsuitable for use as a therapeutic agent in MS (Panitch, Hirsch, Schindler, & Johnson, 1987). The interferons also differ with respect to their cellular sites of synthesis. Interferon beta is produced by various cell types including fibroblasts and macrophages. Natural interferon beta is glycosylated and has a single N‐linked complex carbohydrate moiety. Glycosylation of other proteins is known to affect their stability, activity, bio‐distribution, and half‐life in blood. However, the effects of interferon beta that are dependent on glycosylation are not fully defined. Interferon gamma is induced by the stimulation of sensitized lymphocytes with antigen or non‐sensitized lymphocytes with mitogens.

**Figure 1:**
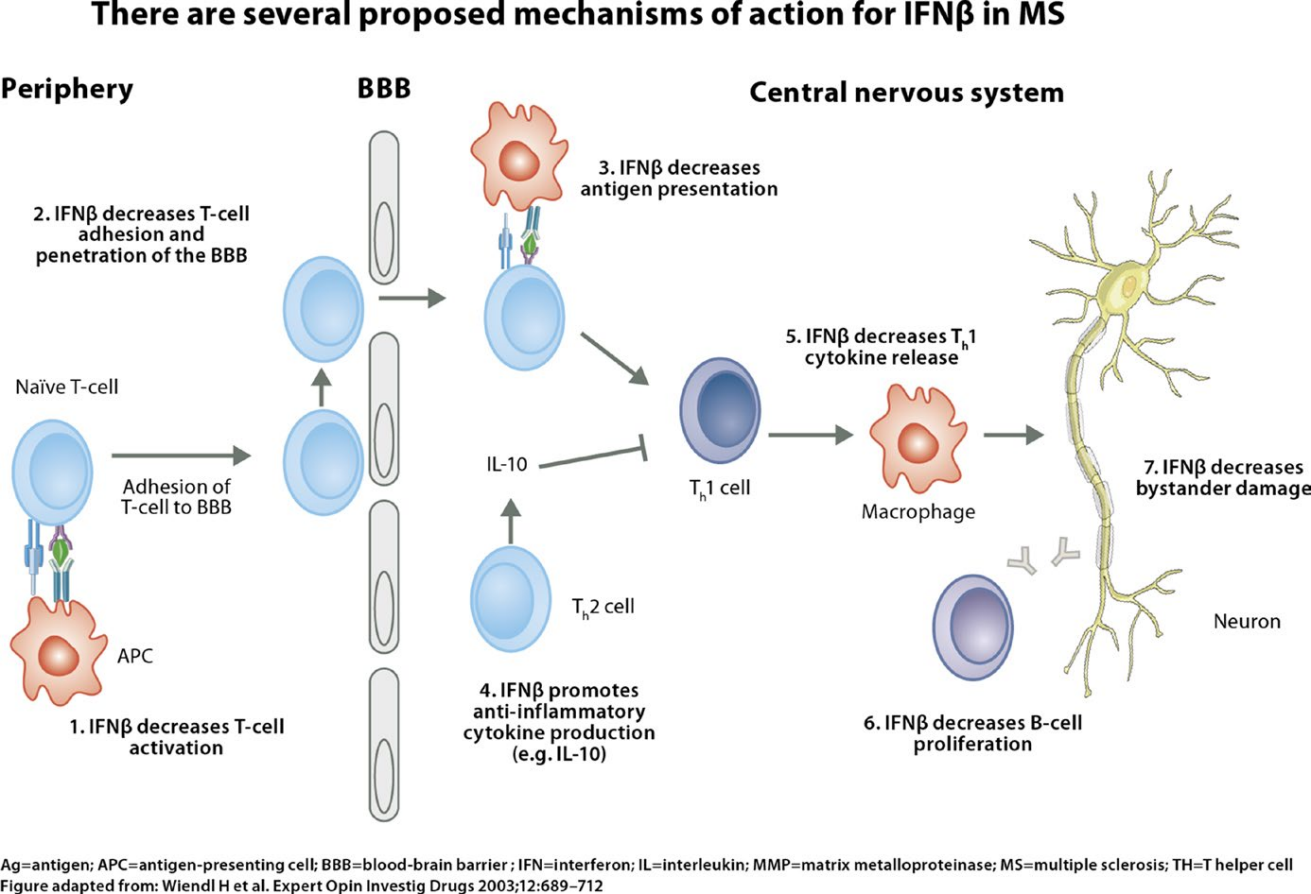
Mechanisms of action for IFNβ in multiple sclerosis. Interferon beta exerts its biological effects by binding to specific receptors on the surface of human cells. This binding initiates a complex cascade of intracellular events that leads to the expression of numerous interferon‐induced gene products and markers (Wiendl & Kieseier, 2003)

The mechanism of action of interferon beta is complex, involving effects at multiple levels of cellular function. Interferon beta appears to directly increase expression and concentration of anti‐inflammatory agents while downregulating the expression of proinflammatory cytokines. Interferon beta exerts its biological effects by binding to specific receptors on the surface of human cells. This binding initiates a complex cascade of intracellular events that leads to the expression of numerous interferon‐induced gene products and markers. These include MHC Class I, Mx protein, 2′/5′‐oligoadenylate synthetase (OAS), β2‐microglobulin, and neopterin. Some of these products have been measured in the serum and cellular fractions of blood collected from patients treated with interferon beta (Figure 1).

After a single intramuscular (IM) dose of interferon beta‐1a, serum levels of these products remain elevated for at least 4 days and up to 1 week (Wiendl & Kieseier, 2003). Whether the mechanism of action of interferon beta in MS is mediated by the same pathway as the biological effects described here is not known.

Increased amounts of neopterin are produced by human monocytes/macrophages upon stimulation of interferon beta. Measurement of neopterin concentrations in body fluids like serum, cerebrospinal fluid or urine, provides information about activation of T‐helper cell 1 derived cellular immune activation (Murr, Widner, Wirleitner, & Fuchs, 2002) and pharmacological activity of interferon betas is assessed by serum concentrations of neopterin as a well‐characterized biomarker induced by interferon beta‐1a and peginterferon beta‐1a (Bagnato, Durastanti, Finamore, Volante, & Millefiorini, 2003; Hu et al., 2011). A small study furthermore suggests that neopterin may be considered a useful biomarker of interferon beta responsiveness (Casoni et al., 2004). 2′5′ OAS is also used as an established biomarker of interferon beta activity (Scagnolari et al., 2007).

## 3 Assessments of Benefits and Risks

Multiple sclerosis treatment has become widely differentiated and individually tailor‐made with several treatment alternatives with various modes of action and administration. Still, interferon betas remain the choice of treatment for many patients. In particular, in patients who value the long‐term safety profile of interferon betas and for young females the interferon betas may provide an attractive alternative to the second and third generation MS treatments, which are contraindicated before or at the occurrence of pregnancy (Amato & Portaccio, 2015).

The consistent long‐term safety data on interferon betas have over the years added to a favorable benefit/risk profile. The most frequently occurring adverse effects with interferon betas are injection site reactions and flu‐like symptoms. Injection site reactions occur frequently for subcutaneous (SC) injections but less frequently for IM injections. Flu‐like symptoms tend to be most pronounced at the initiation of the treatment and wears off over time. Flu‐like symptoms may be countered or reduced by symptomatic treatment with nonsteroidal anti‐inflammatory drugs or paracetamol, and additionally dose titration recommendations may apply to the individual interferon betas for improved tolerability (SmPC Avonex, 2016; SmPC Betaferon, 2016; SmPC Plegridy, 2014; SmPC Rebif, 2015).

Several interferon betas are approved and commercially available for the treatment of RRMS, and the individual products differ in formulation, dosing, administration, and benefits/risk profiles including the risk of developing neutralizing antibodies.

Table 1 offers an overview of licensed interferon betas for the treatment of RRMS.

**Table 1:** Commercially available interferons approved for the treatment of RRMS

Product	Active substance	Adm.	Dosing	PK assessments	Qualitative composition	Neutralizing antibodies	Reduction of the annual attack rate (ITT)^a^	Reduction of disability progression (EDSS)^b^	Common adverse effects		References
									Flu‐like symptoms	Injection site reactions	
Plegridy^®^	Pegylated IFN beta‐1a	SC	125 μg/2 weeks	*T*½: 78 ± 15 hr *T*_max_: 1–1.5 days (SmPC Plegridy)	Recombinant from CHO‐K1 covalently linked with methoxy‐polyethyleneglycol (SmPC Plegridy)	<1% (SmPC Plegridy)	36% (Calabresi et al., 2014)	38% (12 w‐CDP; Calabresi et al., 2014) 54% (24 w‐CDP; SmPC Plegridy, 2014)	47% (SmPC Plegridy, 2014)	66% (SmPC Plegridy, 2014)	SmPC Calabresi et al. (2014) Kieseier et al. (2014)
Betaferon^®^/Extavia^®^	IFN beta‐1b	SC	250 μg/every other day	*T*½: 5 hr *T*_max_: 1–8 hr Bioavailability: ~50% (SmPC Betaferon)	Recombinant from *Escherichia coli* (SmPC Betaferon)	23%–41% (SmPC Betaferon)	30% (The IFNB Multiple Sclerosis Study Group, 1993)	31% (The IFNB Multiple Sclerosis Study Group, 1993) (12 w‐CDP; SmPC Betaferon, 2016) NS	52% (SmPC Betaferon, 2016)	85% (SmPC Betaferon, 2016)	SmPC The IFNB Multiple Sclerosis Study Group (1993)
Avonex^®^	IFN beta‐1a	IM	30 μg weekly	*T*½: 10 hr *T*_max_: 5–15 hr Bioavailability: ~40% (SmPC Avonex)	Recombinant from CHO‐K1 (SmPC Avonex)	5%–8% (SmPC Avonex)	32% (Jacobs et al., 1996)	37% (24 w‐CDP; Jacobs et al., 1996)	61% (Jacobs et al., 1996)	15% (Jacobs et al., 1996)	SmPC Jacobs et al. (1996) Rudick et al. (1997)
Rebif^®^	IFN beta‐1a	SC	22 μg 3 × weekly 44 μg 3 × weekly	Apparent *T*½: 50–60 hr *T*_max_: 8 hr (SmPC Rebif, 2015)	Recombinant from CHO‐K1 (SmPC Rebif, 2015)	24% (SmPC Rebif, 2015) 13‐14% (SmPC Rebif, 2015)	27% (PRISMS Study Group, 1998) 33% (PRISMS Study Group, 1998)	30% (12w‐CDP; SmPC Rebif, 2015) 39% (12w‐CDP; SmPC Rebif, 2015)	70% (SmPC Rebif, 2015)	30% (SmPC Rebif, 2015)	SmPC PRISMS Study Group, (1998)

The data are extracted from the respective summary of product characteristics and the pivotal registration studies performed in RRMS. IFN, Interferon; *T*½, half‐life assessed with neopterin; *T*_max_, time to peak concentration assessed with neopterin; CHO‐K1, Chinese hamster ovary cells; ITT, intention‐to‐treat. SC, subcutaneous; IM, intramuscular ITT, intention‐to‐treat analysis; CDP, confirmed disability progression. Data in the columns Active substance, Administration, Dosing, PK assessments, Qualitative composition and neutralizing antibodies are collected from the respective Summary of Product Characteristics (SmPC). NS, not significant.

a Compared to placebo.

b Proportion with ≥1‐point progression on EDSS.

Heterogeneity of the disease and the natural—progressive—course of RRMS may lead to periods of disease activity and the question is whether this may be caused by lack of response to the therapy. Clinically, this calls for analysis of neutralizing antibodies. The development of neutralizing antibody activity suggests a reduction in clinical efficacy, and there is an evident rationale for therapy switch or treatment discontinuation (Sorensen et al., 2003). With the expanded panel of treatment this may be less of an issue for most patients, however, in young women who wish to become pregnant, this may be a challenge, particularly if they have previously developed neutralizing antibodies, since interferon betas are considered the safer treatment choice in this cohort of patients. Thus, there is still a mandate to consider the risk of development of neutralizing antibodies when evaluating therapy options.

## 4 Innovative Formulations Counter Adherence Issues

Interferon beta is a part of the first‐line treatment of MS and is injected intramuscularly or subcutaneously. Administration may be associated with discomfort from the injection, in addition to potential adverse effects following dosing. The proportion of patients who do not follow the treatment according to the prescription (i.e., is nonadherent) in injectable therapies for MS is estimated to be 15%–60% (Costello, Kennedy, & Scanzillo, 2008; Devonshire et al., 2011; Menzin et al., 2013). Notwithstanding the nature of the disease, nonadherence is a significant problem for the individual patient who does not achieve full effect of the treatment (Sabate & De, 2004), and increases the risk of relapses (Steinberg, Faris, Chang, Chan, & Tankersley, 2010). Additionally, nonadherence constitutes an economic burden to society in the treatment of MS, risking that the underlying disease progresses more rapidly (Steinberg et al., 2010).

**Figure 2:**
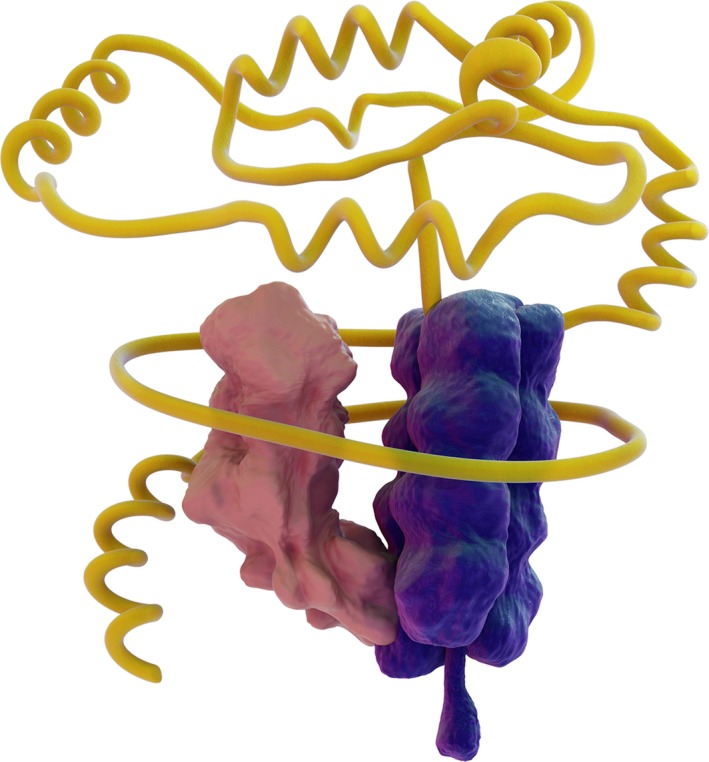
Pegylation implies addition of a polyethylene chain to an interferon beta‐1a molecule

More recently, interferon beta has been modified via pegylation resulting in a prolonged half‐life of the active substance while the dosage interval is extended, allowing for fewer injections, less accumulated discomfort and improved adherence of treatment (Figure 2). Pegylation stabilizes the molecule chemically by protecting it from degradation and proteolysis (Kang, Deluca, & Lee, 2009). The increased circulation time improves bioavailability and prolongs the half‐life of the active substance, implying a potential beneficial impact on efficacy (Hu et al., 2015).

The slower degradation has also been shown to decrease the probability of developing neutralizing antibodies (Kang et al., 2009). Altogether, the changed properties and pharmacodynamic effects of PEGylated interferon (peginterferon) represent a new era of DMTs for RRMS (Table 1).

## 5 Pharmacokinetic and Pharmacodynamic Profile of Peginterferon Beta

Compared to interferon beta‐1a 30 μg IM the half‐life of peginterferon beta‐1a 125 μg SC is longer (10 hr and 2–3 days, respectively; Hu, Miller, & Richman, 2012; Hu et al., 2015; SmPC Avonex, 2016). The serum concentration of peginterferon beta‐1a appears to be dose proportional in the range of 63–188 μg as observed in a single and multiple dose study in healthy subjects (Hu et al., 2012). The pharmacokinetics observed in people with MS was similar to the pharmacokinetics in healthy subjects (Hu et al., 2011, 2012, 2015).

In a phase 1 study of peginterferon beta‐1a multiple doses were administered subcutaneously with dosing intervals of 2 (Q2W) and 4 (Q4W) weeks. Biomarkers shown in these clinical trials demonstrated a stronger and prolonged response in administration of peginterferon beta‐1a than by non‐pegylated interferon beta1a (Hu et al., 2012). Subcutaneous peginterferon beta‐1a resulted in ninefold higher exposure (AUC_168 hr_) 3.5‐fold higher *C*_max_, following single doses of 125 μg(12 MIU), compared to IM administration of 30 μg (6 MIU) non‐pegylated beta‐1a. The extended dosing interval with peginterferon beta‐1a gave no unexpected adverse events, no signs of accumulation of peginterferon beta‐1a and no loss of pharmacological properties at repeated dosing (Hu et al., 2012). Pegylation resulted in increased activity *in vivo*, resulting in an extended half‐life and increased bioavailability.

Recently, a comparative pharmacokinetic study demonstrated that one dose of SC peginterferon beta‐1a delivered significantly greater drug exposure than SC interferon beta‐1a, 44 μg three times a week, over 2 weeks and a lower frequency of adverse events (AEs) (Hu et al., 2016). The COMPARE study was an open‐label, crossover, pharmacokinetic study evaluating drug exposure and the safety and tolerability of SC peginterferon beta‐1a, 125 μg and SC interferon beta‐1a, 44 μg three times a week, over 2 weeks in healthy subjects. Thirty healthy subjects received one dose of peginterferon beta‐1a (125 μg SC) or six doses of interferon beta‐1a (44 μg SC) over 2 weeks, followed by the alternate treatment after a 2‐week washout period. Drug concentrations were measured using an enzyme‐linked immunosorbent assay and PK parameters including cumulative area under the concentration time curve (AUC_336 h_r) over 2 weeks and maximum observed serum concentrations (*C*_max_) were estimated using a non‐compartmental analysis. The PK analysis population comprised 26 subjects for each treatment. *C*_max_ was 3.5‐fold higher with one dose of peginterferon SC (944 pg/ml) than after six doses of interferon beta SC (266 pg/ml) (Hu et al., 2016). Drug exposure (AUC_336 h_r) was 60% higher with SC peginterferon beta‐1a than with SC interferon beta‐1a (117.4 [95% confidence interval 95.6–144.3] hr·ng/ml vs. 73.1 [61.2–87.3] hr·ng/ml, respectively; *p* < .0001) (Hu et al., 2016). Injection‐site reactions (ISRs) were the most common AEs observed with both treatments. Numerically lower frequencies and incidence rates of ISRs, headache, myalgia, and chills, were observed with SC peginterferon beta‐1a (Hu et al., 2016).

Due to the reduced number of injections that peginterferon beta‐1a offers, the frequencies of injection site reactions and flu‐like symptoms were numerically higher with SC interferon‐beta‐1a treatment compared with SC peginterferon beta‐1a treatment (Hu et al., 2016).

**Figure 3:**
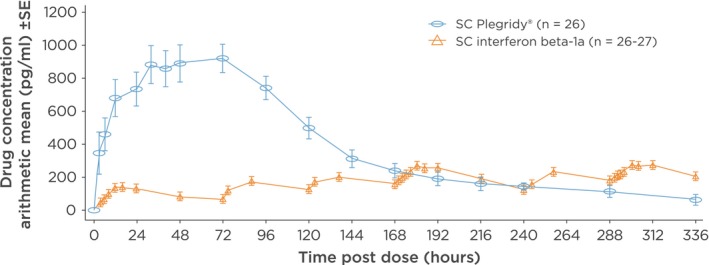
Peginterferon beta‐1a yields a higher overall drug exposure compared to subcutaneous (SC) interferon beta‐1a. The curve demonstrates a higher overall drug exposure with peginterferon beta‐1a compared to SC interferon beta‐1a (Hu et al., 2016)

Conclusively, the study demonstrated that peginterferon beta‐1a provided significantly greater drug exposure, following a single dose, compared to six doses of SC interferon beta‐1a over 2 weeks. Moreover, higher drug exposure was not associated with increased incidence of side effects; peginterferon beta‐1a demonstrated an improved tolerability profile with respect to injection site reactions and flu‐like symptoms (Figure 3).

## 6 Clinical Effects of Peginterferon Beta‐1a

In the ADVANCE study, a 2‐year double‐blind, parallel group, phase 3 study enclosing a placebo controlled design for the first 48 weeks, peginterferon beta‐1a given Q2W significantly reduced the relapse rate compared with placebo (Calabresi et al., 2014). The adjusted annualized relapse rates (ARR) were 0.397 (95% CI 0.328–0.481) in the placebo group versus 0.256 (0.206–0.318) in Q2W group (rate ratio for Q2W 0.644, 95% CI 0.500–0.831, *p* = .0007), corresponding to a relative risk reduction in ARR of 36% at Year 1 (Calabresi et al., 2014). Peginterferon beta‐1a reduced 12‐week and 24‐week confirmed disability progression by 38% (*p* = .0383) versus placebo at Year 1 (Calabresi et al., 2014); while the 24‐week confirmed disability progression for the peginterferon beta‐1a group was 54% (*p* = .0069; SmPC Plegridy, 2014).

In the second year of ADVANCE, all placebo patients were re‐randomized to either peginterferon beta‐1a Q2W or Q4W (Kieseier et al., 2014). Compared to Year 1, the ARR was further reduced in Year 2 with Q2W dosing (Year 1: 0.230 [95% CI 0.183–0.291], Year 2: 0.178 [0.136–0.233]; Kieseier et al., 2014). Patients starting peginterferon beta‐1a Q2W from Year 1 displayed improved efficacy versus patients initially assigned placebo, with reductions in ARR (37%, *p* < .0001), risk of relapse (39%, *p* < .0001), 12‐week disability progression (33%, *p* = .0257), and 24‐week disability progression (41%, *p* = .0137; Kieseier et al., 2014).

83% of patients taking placebo and 94% of patients taking peginterferon beta‐1a Q2W reported adverse events including relapses. The most common adverse events associated with peginterferon beta‐1a were injection site reactions, flu‐like symptoms, pyrexia, and headache. 76 (15%) patients taking placebo, 11% of patients taking study drug Q2W reported serious adverse events; relapse, pneumonia, and urinary tract infection were the most common; with apparently fewer flu‐like symptoms in comparison, the overall safety profile of peginterferon beta‐1a seems to resemble that of other interferon beta therapies (Calabresi et al., 2014; Kieseier et al., 2014).

In the ADVANCE study 1332 (88%) of 1,512 participants completed the first year of the study. Treatment adherence—defined as the number of doses a patient received divided by the number they were expected to have received—was greater than 99% in each treatment group (Calabresi et al., 2014).

Although direct head‐to‐head studies have not been conducted, the data suggest that the improved PK/PD profile of peginterferon beta‐1a may confer comparable clinical effects as non‐pegylated interferon beta, but with a formulation that offers prolonged injection intervals. Patients thus have an effective interferon beta treatment option that also significantly reduces the number of injections, from up to 156 to just 26 per year with a minimum risk of developing of neutralizing antibodies (Calabresi et al., 2014; Kieseier et al., 2014). Furthermore, this feature improves the user‐friendliness of the therapy, allowing a significantly lower number of injections.

## 7 Magnetic Resonance Imaging

Post hoc analyses of the efficacy of peginterferon beta‐1a Q2W versus Q4W were conducted for clinical and MRI endpoints over 2 years. Over 2 years, peginterferon beta‐1a Q2W produced favorable MRI outcomes compared with peginterferon beta‐1a Q4W (Kieseier et al., 2014). The results enclose a T2‐weighted lesion mean ratio Q2W versus Q4W of 0.40 (95% CI 0.32, 0.49) *p* < .0001) and a percentage reduction of gadolinium enhanced lesions at 2 years in the group of Q2W versus Q4W: 71% reduction; *p* < .00001.

## 8 Future Prospects

Interferon beta is a treatment that has been used for many years in MS, however, developments in this efficacious treatment have continued over the years, with pegylation as the most recent innovation. The data on peginterferon beta‐1a have demonstrated this new interferon beta to be an effective treatment with an attractive benefit/risk profile and with a markedly lessened injection burden compared to non‐PEGylated formulations (Calabresi et al., 2014; Kieseier et al., 2014). With improved innovative formulations, there is a possibility that efficacy, administration frequency, and adherence rates may improve.

## Conflict of Interest

The author has acted as board member of national advisory boards for Roche and Biogen.
